# Exploiting Nest Defense Behavior of Hosts: A Case Study of Successful Parasitism by a Common Cuckoo

**DOI:** 10.1002/ece3.71704

**Published:** 2025-06-27

**Authors:** Qiqi Liu, Wei Liang

**Affiliations:** ^1^ Ministry of Education Key Laboratory for Ecology of Tropical Islands College of Life Sciences, Hainan Normal University Haikou China

**Keywords:** brood parasitism, common cuckoo, gray bushchat, host activity hypothesis, host nest defense behavior

## Abstract

In this study, we document the case of a common cuckoo ( *Cuculus canorus* ) successfully locating and parasitizing the nest of a gray bushchat ( *Saxicola ferreus* ) by exploiting the latter's nest defense behavior in Liuzhi, Guizhou, southwest China. We found that the common cuckoo could identify the secluded nest site by observing the host's defense behaviors (e.g., frequent lunging and emitting alarm calls), and successfully laid its egg in the nest despite aggressive host defense. This phenomenon provides strong support for the “host activity hypothesis.” The nest defense behavior exhibited by the gray bushchat aimed at protecting its nest, inadvertently provided cues to the common cuckoo regarding its nest site. This finding reveals a complex coevolutionary relationship between parasitism and antiparasitism, highlighting the dual role of the host's nest defense behavior in both reducing brood parasitism and providing parasites with nest‐site cues. Our findings offer a new perspective for further exploring the behavioral strategies of parasitic birds and the evolutionary dynamics of the host's nest defense behavior.

## Graphical abstract

We document the case of a common cuckoo ( *Cuculus canorus* ) successfully locating and parasitizing the nest of a gray bush chat ( *Saxicola ferreus* ) by exploiting the latter's nest defense behavior. Our findings offer a new perspective for further exploring the behavioral strategies of parasitic birds and the evolutionary dynamics of the host's nest defense behavior.

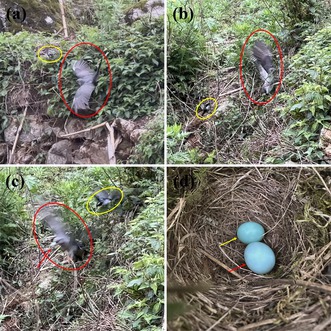

## 1 Introduction

Parasitic birds rely on host nests for reproduction, and the key to their successful parasitism is the ability to accurately locate host nests. The means by which parasitic birds locate host nests has remained an interesting topic in behavioral ecology research. In general, two main hypotheses have been proposed to explain how parasitic birds locate host nests: the habitat search and host activity hypotheses (Davies 2000). The habitat search hypothesis posits that parasites may find nest sites by actively searching for nests in suitable habitats, whereas the host activity hypothesis suggests that parasitic birds find nest sites by observing the behavioral activities of their hosts. Previous studies have shown that host activity and defense behavior may provide parasitic birds with critical cues for nest sites. For example, the brown‐headed cowbird ( *Molothrus ater* ) triggers host defensive responses when searching for nests to parasitize. Their nest‐searching behaviors include walking on the ground, flying or perching near the nest, and observing host activities, such as nest building (Norman and Robertson 1975; Thompson and Gottfried 1976, 1981; Gates and Gysel 1978). Some scholars have conducted studies on the nest parasitism behavior of the brown‐headed cowbird by setting up real but unguarded nests of adult birds to observe its parasitism. Their results showed that the experimental nests were not parasitized in the absence of host activity, implying that host activity is vital for eliciting nest parasitism in the brown‐headed cowbird (Robinson and Robinson 2001). Similarly, Soler and Pérez‐Contreras (2012) tested the effect of magpie ( *Pica pica* ) activity on nest‐searching by the great spotted cuckoo ( *Clamator glandarius* ). Their results showed that before egg‐laying, natural, active magpie nests were parasitized significantly more frequently than those without eggs or parental activity. However, the nest defense behaviors of hosts may also influence the parasitism success of cuckoos. As previously documented in a common cuckoo ( *Cuculus canorus* ) host, the Oriental reed warbler ( *Acrocephalus orientalis* ) mobbed the cuckoo upon discovery, thus effectively resisting its parasitism (Wang et al. 2020, 2023). Therefore, whether the common cuckoo follow the activities of its host to locate target nests needs further investigation.

The gray bushchat ( *Saxicola ferreus* ) is a common cuckoo host in our study area, Liuzhi (26°13′ N, 105°42′ E; with an elevation of 1070–1657 m), Guizhou, southwest China (Zhong et al. 2023), and this area has a typical karst landscape, with the majority of gray bushchat nests being found in rocky holes, ground ridges, road ridges, or pits (Zhong et al. 2023). The nest sites are relatively secluded and may not be easily discovered by the common cuckoo (Zhong et al. 2023). In addition, nest defense behaviors of the gray bushchat differed from those of the Oriental reed warbler (Wang et al. 2023), as in the gray bushchat, nest defense is usually undertaken by the male, without cooperative defense between individuals (Zhong et al. 2023). In this study, we document the case of a common cuckoo successfully locating and parasitizing the nest of a gray bushchat by exploiting the latter's nest defense behavior.

## 2 Observations and Results

**FIGURE 1:**
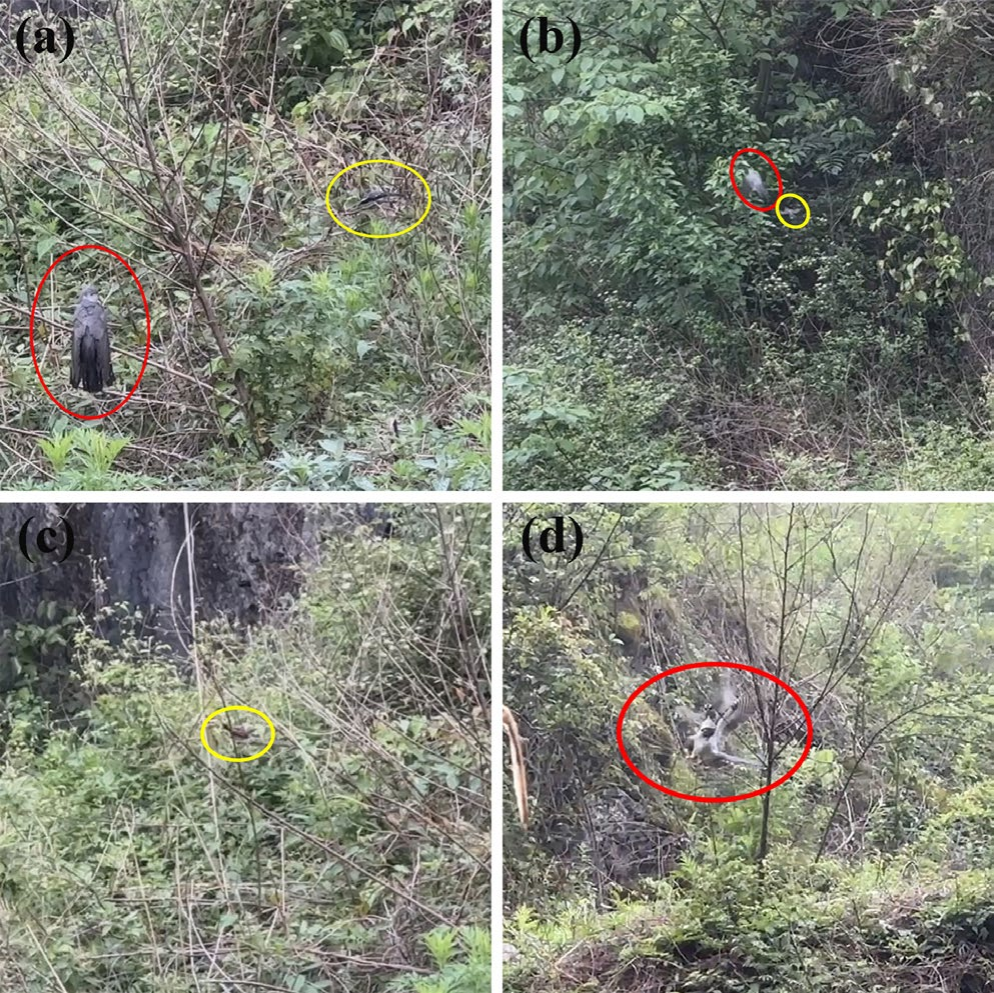
Nest defense behaviors of the gray bushchat ( *Saxicola ferreus* ) against the common cuckoo ( *Cuculus canorus* ) near the nest site. The letter (a) refers to upon discovering the common cuckoo, the male gray bushchat emits an alarm call and assumes an aggressive posture (the red and yellow circles indicate the common cuckoo and male gray bushchat, respectively; see Video S1 for details). The letter (b) refers to the male gray bushchat chasing and driving away the common cuckoo (the red and yellow circles indicate the common cuckoo and male gray bushchat, respectively; see Video S2 for details). The letter (c) refers to the female gray bushchat watching around the nest and emitting alarm calls but not participating in driving away the common cuckoo (the yellow circle indicates the female gray bushchat; see Video S2 for details). The letter (d) refers to the male gray bushchat attacking the common cuckoo on the head (the red circle indicates the common cuckoo being attacked on the head by the male gray bushchat; see Video S3 for details).

**FIGURE 2:**
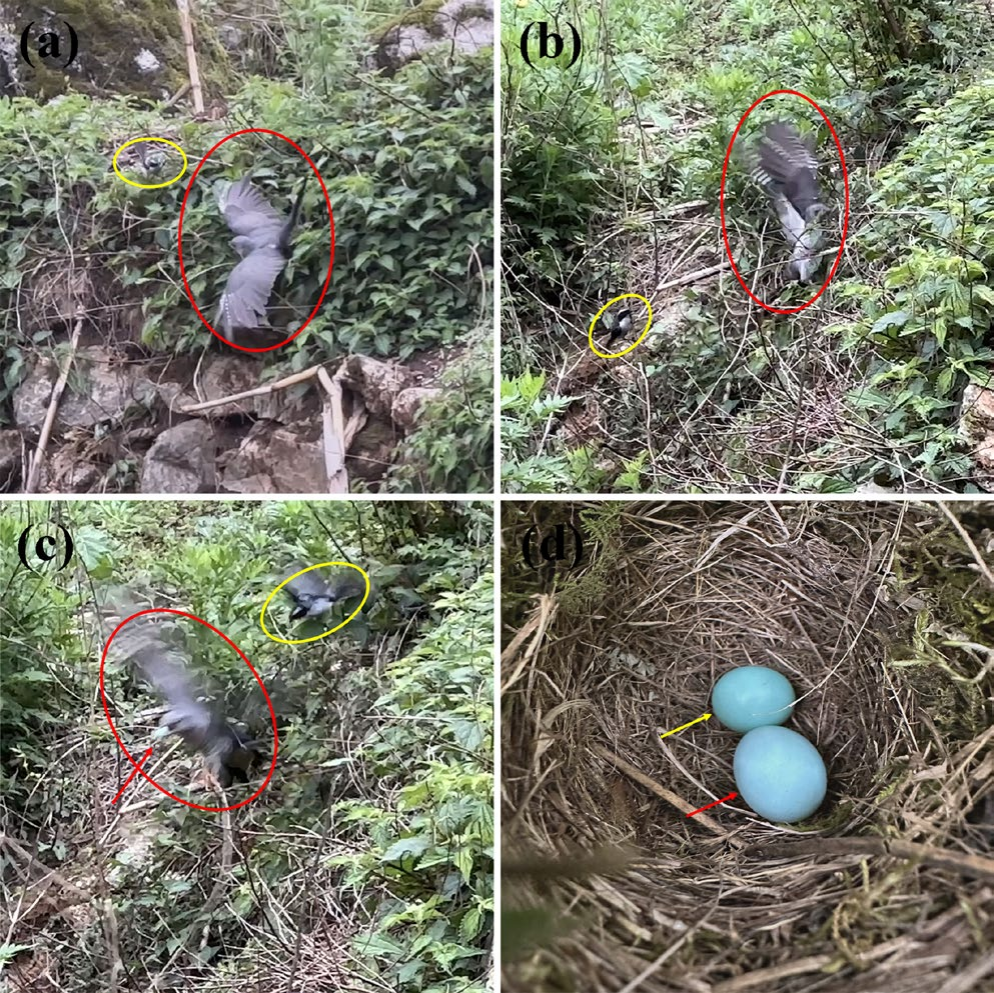
Accurate discovery and parasitism of the gray bushchat nest by the common cuckoo. The letter (a) refers to the common cuckoo failing to correctly locate the nest site, while the male gray bushchat watching (the red and yellow circles indicate the common cuckoo and male gray bushchat, respectively; see Video S4 for details). The letter (b) refers to the common cuckoo identifying the correct nest site and entering the nest to lay its egg despite attacks by the male gray bushchat. The male gray bushchat remains highly vigilant in the vicinity and attacks the common cuckoo several times (the red and yellow circles indicate the common cuckoo and male gray bushchat, respectively, and the red arrow points to the correct nest site where the common cuckoo enters; see Video S5 for details). The letter (c) refers to the common cuckoo leaving the nest while carrying a host egg after laying its egg, while the male gray bushchat continues to attack the cuckoo (the red and yellow circles indicate the common cuckoo and male gray bushchat, respectively, and the red arrow points to the host egg held by the common cuckoo; see Video S5 for details). The letter (d) refers to eggs of the common cuckoo and gray bushchat in the latter's nest (the red arrow points to the common cuckoo egg).

On May 2, 2024, at 15:22 (Beijing local time, UTC + 8), along a rural road in the study area, we found that a common cuckoo with gray dorsal coloration successfully found a secluded nest site by observing the host's defense behaviors (e.g., frequent lunging and emitting alarm calls), and successfully laid its egg despite the host's aggressive defense. (1) Initial observation phase. The common cuckoo when it was perched on a leafy branch approximately 15 m from the nest of the gray bushchat. Its presence elicited an alarm call from a brown‐breasted bulbul ( *Pycnonotus xanthorrhous* ) perched on the same tree; however, despite the persistent calls of the brown‐breasted bulbul, the common cuckoo remained perched on the branch while repeatedly looking downward. (2) Approaching the nest and host defense. Once the brown‐breasted bulbul flew away, the common cuckoo, after constant vigilance, descended to a dead branch of a shrub near the nest, where it perched and continued watching. After 4 s, a male gray bushchat flew toward the common cuckoo and perched itself on a dead branch 1 m away from the common cuckoo. This male gray bushchat lowered its body and tail, extended its neck, and displayed an aggressive posture toward the common cuckoo while emitting an alarm call (Figure 1a details in Video S1). (3) First conflict and attempt. When the common cuckoo took flight, the male gray bushchat attacked it, constantly pecking at the common cuckoo with its beak. The common cuckoo also retaliated with its mouth wide open but was eventually forced to perch on a branch near the nest. The gray bushchat followed closely behind and perched on the same branch, just 1 m away from the common cuckoo, where it emitted alarm calls and returned to the vicinity of its nest. After 1 min, the common cuckoo subsequently flew to a shrub in close proximity to the nest, where it was intercepted by the male gray bushchat adjacent to the nest, and the latter chased the common cuckoo while emitting alarm calls. During this period, a female gray bushchat flew from a nearby shrub to a dead branch near the nest to observe and emit alarm calls. However, it did not join the male in chasing the common cuckoo (Figure 1b,c; details in Video S2). (4) Multiple attempts and egg‐laying. Instead of fleeing when chased, the cuckoo circled the nest to test its approach and perched nearby again to observe. The male gray bushchat repeatedly swooped down to peck at the cuckoo's head (Figure 1d; details in Video S3). After briefly leaving the nest area, the cuckoo returned once more. Although it did not find the correct nest, it assumed an egg‐laying posture, with its wings spread out, body lowered, head facing outwards, and tail feathers held high. The male gray bushchat renewed vigilance forced it to abort the action (Figure 2a, details in Video S4). After several attempts, the common cuckoo accurately located the host nest. Despite continuous attacks by the male gray bushchat and strong alarm calls from the female gray bushchat, it successfully completed egg‐laying in about 7 s (Figure 2b) and fled carrying one of the host's eggs (Figure 2c; details in Video S5).

The parasitic cuckoo egg was solid blue in color, closely mimicking the appearance of the host egg (Figure 2d). The weight of the parasitic egg was 4.06 g, with dimensions of 24.25 × 19.14 mm. The parasitized nest of the gray bushchat was located on a slope with a gradient of 32°, with a size of 8.4 × 5.3 × 5.2 cm in nest outer diameter, inner diameter, and nest depth, respectively.

## 3 Discussion

In this study, we observed and video‐documented the case of a common cuckoo using nest defense behaviors of the gray bushchat to successfully locate and parasitize its nest, which provides strong support for the “host activity hypothesis.” Our finding suggests that parasitic cuckoos can locate nest sites by exploiting host defense behaviors (e.g., frequent lunging, emitting alarm calls) to locate secluded nest sites. This behavior may be an adaptive strategy developed by the common cuckoo over evolutionary process, enabling it to achieve successful parasitism despite the strong host defenses. Furthermore, this study provides additional evidence that nest defense behaviors may not always effectively prevent brood parasitism, as suggested by Wang et al. (2023): when the number of host birds is ≤ 2, they become virtually incapable of resisting cuckoo parasitism despite their defensive efforts.

Nest defense behavior is commonly perceived as the host's first line of defense against parasites (Feeney et al. 2014). For example, the Oriental reed warbler exhibits extremely high levels of aggression toward the common cuckoo when defending its nest (Ma et al. 2018; Wang et al. 2023). However, in some cases, this defense behavior may not work well. As demonstrated by the results of this study, the nest defense behavior of the gray bushchat, while intended to protect the nest from predators or parasites, actually provided the common cuckoo with critical cues regarding its nest. The common cuckoo could identify the nest site by observing the host's defense behavior, and successfully laid its egg despite the host's aggressive defenses. This phenomenon suggests that under specific circumstances, the host's nest defense behavior may also play a role in facilitating successful parasitism by parasitic birds. Moreover, this phenomenon suggests a complex coevolutionary relationship between parasitism and anti‐parasitism behaviors. Some host birds respond to parasitic pressure by adjusting the intensity of their defense behavior; however, these adjustments may not consistently prove to be successful. For example, the cowbird uses the intensity of its host's defensive responses toward them as a cue to locate potential nests for parasitism; hosts with more aggressive responses provide more cues regarding nest site than those without them; thus, the former may be more frequently parasitized (Robertson and Norman 1977). Thus, the host's nest defense behavior exhibits a dual effect of evolutionary trade‐off: while such defensive behaviors may reduce parasitism success, the very act of defense may simultaneously increase parasitism risk by revealing nest location cues.

In conclusion, this study is the first to document an actual case of a common cuckoo successfully locating and parasitizing a host nest using the host's nest defense behavior. This finding reveals the complex adaptive strategies that parasitic birds have developed over the course of evolution, as well as highlights the dual role of host defense behavior in the coevolutionary dynamics. Future studies should further explore the mechanisms by which cuckoos recognize and exploit the nest defense behaviors of hosts, as well as the extent to which the exploitation and elicitation of host defensive behavior by the cuckoo may subsequently influence host egg rejection behavior. Moreover, investigating the effects of the host's nest defense behavior on parasitism success will contribute to an enhanced understanding of the evolutionary dynamics between parasitism and anti‐parasitism behaviors.

## Author Contributions

**Qiqi Liu:** data curation (equal), formal analysis (equal), investigation (equal), writing – original draft (equal). **Wei Liang:** conceptualization (equal), funding acquisition (lead), supervision (lead), validation (equal), writing – review and editing (lead).

## Ethics Statement

The experiments comply with the current laws of China. No special permit was required for this study as it was not involved in animal or plant collection.

## Conflicts of Interest

The authors declare no conflicts of interest.

## Supporting information


**Video S1.** The male gray bushchat ( *Saxicola ferreus* ) emits alarm calls and assumes an attack posture upon discovering the common cuckoo ( *Cuculus canorus* ).

**Video S2.** The male gray bushchat chases and drives away the common cuckoo.

**Video S3.** The male gray bushchat confronts the common cuckoo and attacks its head.

**Video S4.** The common cuckoo fails to accurately locate the nest site while the male gray bushchat continues to watch.

**Video S5.** The common cuckoo finds and enters the correct nest site to lay its egg despite attacks by the male gray bushchat. The male gray bushchat remains highly vigilant in the vicinity and attacks the common cuckoo several times. After laying an egg, the common cuckoo escapes the nest while carrying a host egg.

## Data Availability

No data used for this study. Videos are provided as (Videos S1–, S5) and can be found at https://figshare.com/s/2a762b7dc8ce051e69e5 (https://doi.org/10.6084/m9.figshare.28644941).
